# A Rare Case of Rhabdosarcoma on the Upper Pole of the Kidney

**DOI:** 10.7759/cureus.60010

**Published:** 2024-05-09

**Authors:** Kovvuru Ashrita, Korra R Naik, Lokesh Ethuri, Sanjana Nelogal, Aisha Reshie, Sindhu C Valiveti, Mihirkumar P Parmar

**Affiliations:** 1 Internal Medicine, Osmania Medical College, Hyderabad, IND; 2 Internal Medicine, JJM Medical College, Bangalore, IND; 3 Internal Medicine, Government Medical College Srinagar, Srinagar, IND; 4 Internal Medicine, SVIMS-SPMCW (Sri Venkateswara Institute of Medical Sciences-Sri Padmavathi Medical College for Women), Tirupati, IND; 5 Internal Medicine, Gujarat Medical Education and Research Society, Vadnagar, IND

**Keywords:** surgical excision, multimodal treatment, pediatric renal tumors, renal rhabdomyosarcoma, rhabdomyosarcoma

## Abstract

Rhabdomyosarcoma arising from the upper pole of the kidney is an exceedingly rare occurrence, with only a few documented cases reported in the literature. Here, we present a case of a one-year-old Indian male child diagnosed with rhabdomyosarcoma localized to the upper pole of the kidney. The patient presented with abdominal distension persisting for three days, and imaging studies revealed a mass consistent with renal sarcoma. Surgical excision was performed, followed by histopathological examination confirming the diagnosis of rhabdomyosarcoma. Despite aggressive management, the patient's prognosis remains guarded due to the disease's aggressive nature. This case highlights the importance of considering rhabdomyosarcoma in the differential diagnosis of renal masses, particularly in atypical locations. Early diagnosis and a multimodal treatment approach are crucial for optimizing outcomes in such rare cases.

## Introduction

Rhabdomyosarcomas (RMSs) are malignant soft-tissue tumors characterized by skeletal muscle differentiation. They typically arise from chromosomal translocations involving the *PAX3* gene on chromosome 2 and the *FOXO1* gene on chromosome 13, leading to aberrant gene products that promote tumor formation [1]. The overall incidence of RMS in individuals below 20 years of age is 4.58 per million per year in the United States [2], with slight variations across different regions. In Europe, the incidence is slightly higher at 5.4 per million per year [3], while in Japan, it is lower at 3.4 per million per year [3]. Data on the prevalence of this specific presentation within the kidney is limited worldwide, including in India.

RMS primarily affects the pediatric age group, with the average age of presentation being eight years, and it is uncommon after the age of 45 years [4]. It exhibits a slight male predilection and is histologically classified into four subtypes: embryonal, alveolar, spindle cell/sclerosing, and pleomorphic, with embryonal and alveolar types being the most common [4]. The tumor's predilection sites include the head and neck (26%), trunk and other areas (30%), extremities (13%), and the genitourinary tract (12%) [2]. The etiology of RMS remains unclear, although several risk factors have been identified, including in utero radiation exposure, accelerated in utero growth, low socioeconomic status, and parental recreational drug use during pregnancy [5]. Additionally, RMS has been associated with certain familial syndromes, such as neurofibromatosis, Noonan syndrome, Li-Fraumeni syndrome, Beckwith-Wiedemann syndrome, and Costello syndrome [5]. Treatment typically involves a multidisciplinary approach, with chemotherapy as the mainstay, often complemented by surgical resection with or without radiotherapy. Despite advancements in treatment modalities, the overall five-year survival rate for RMS is approximately 68% [2]. Factors associated with a favorable prognosis include early age at presentation (1-9 years), tumor localization in the orbit or genitourinary tract, embryonal subtype, and localized disease [2].

Through this report, we present a rare case of renal RMS in a pediatric patient, highlighting its clinical presentation, radiological features, histopathological characteristics, and therapeutic challenges. We hope to contribute to the existing knowledge base on renal RMS and emphasize the importance of considering it in the differential diagnosis of pediatric renal masses. By documenting and analyzing such cases, we aim to contribute to the existing body of knowledge, enhance clinical awareness, and optimize patient care delivery.

## Case presentation

A one-year-old male patient presented in the outpatient department with abdominal distension persisting for the past three days. Physical examination reveals a soft palpable abdominal mass in the right hypochondriac and lumbar regions extending into the umbilical region. Past medical and family histories were unremarkable and no history of benign or malignant tumors was found. Ultrasonography of the abdomen and pelvis revealed a well-defined heterogeneous echogenic lesion with few hypoechoic areas measuring 9×7.4 cm noted arising from the upper pole of the right kidney.

**Figure 1:**
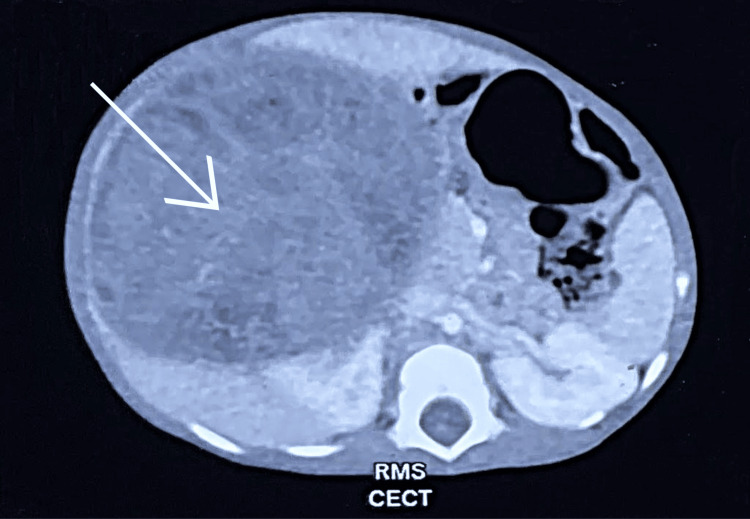
Computed tomography showing right renal mass RMS: Rhabdomyosarcoma; CECT: Contrast-Enhanced Computed Tomography.

Computed tomography (CT) scan of the abdomen and pelvis revealed a well-defined mass lesion measuring 8.5×8.7×8.6 cm involving the right kidney likely arising from upper and mid pole, few non-enhancing cystic areas, and noted infiltrating right renal artery and renal vein (Figure 1). The Radiology differential diagnosis was Wilms tumor. The differences between Wilms tumor and RMS are documented in Table 1.

**Table 1:** Differences between Wilms tumor and RMS RMS: Rhabdomyosarcoma.

Feature	Wilms Tumor	Rhabdomyosarcoma
Cell Type	Nephrogenic blastema (fetal kidney cells)	Myoblasts (muscle cells)
Most Common Sites	Kidneys (almost always unilateral)	Head and neck (orbit, sinuses, nasopharynx), genitourinary (bladder, prostate, vagina), extremities (arms and legs)
Age at Diagnosis	Early childhood (1-5 years old)	Can occur at any age, but is most common in children younger than 10
Gender Predominance	Slightly more common in females	Males are slightly more affected than females
Macroscopic Appearance	Large, well-circumscribed mass	Variable, may be well defined or infiltrative
Microscopic Appearance	Triphasic pattern (epithelial, blastemal, stromal components)	Can show skeletal muscle differentiation (strap cells, cross-striations)
Immunohistochemistry	Positive for WT1 (Wilms Tumor 1 gene product)	Positive for desmin, myogenin, MyoD1 (muscle markers)
Genetics	Associated with mutations in WT1, trisomy 8	Can be associated with genetic syndromes like Li-Fraumeni syndrome, Beckwith-Wiedemann syndrome
Metastasis	Lungs are the most common site	Lungs and other distant sites are common
Treatment	Surgery, chemotherapy, radiation therapy	Surgery, chemotherapy, radiation therapy
Prognosis	Generally good with early diagnosis and treatment	Prognosis depends on stage, histology, and other factors

Ultrasonography-guided renal biopsy of the patient reveals the presence of tissue composed of predominantly round cells with hyperchromatic nuclei with eosinophilic cytoplasm. The patient was put on VAC chemotherapy (vincristine, actinomycin D, cyclophosphamide, mesna) for four cycles.

Post-chemotherapy CT scan of the abdomen and pelvis showed a minimally enhancing hypodense lesion measuring 11×12.6×11.5 cm arising from the upper and midpole of the right kidney. The lesion was displacing the liver superiorly and indenting segments 5 and 6, anterolaterally abutting the anterior abdominal wall and displacing all the bowel loops to the left. The lesion was displacing and compressing the inferior vena cava, posteriorly compressing the right psoas muscle. The patient's tumor showed progression despite chemotherapy. MRI of the abdomen and pelvis reveals a well-defined heterogeneous mixed signal intensity measuring 13×11×11 cm with multiple cystic and hemorrhagic components. The CT scan of the chest and 2D echo showed normal findings. Ultrasonography of the abdomen and pelvis before surgical excision of the tumor revealed a large heterogeneous hyperechoic lesion with anechoic cystic areas in the right kidney measuring 11×7×8 cm. Doppler findings suggested high vascularity in the lesion. The patient underwent a right radical nephrectomy. Post-operative recovery was good. Follow-up after six months showed no evidence of recurrence and metastasis.

The right kidney measured 5×5×4 cm and weighed 500 g. The cross-section revealed a large ill-defined mass with a homogeneous cut surface occupying the upper, middle, and lower poles of the right kidney. Few areas of hemorrhagic and necrosis were seen. Immunohistochemistry studies (IHC) revealed positive tumor cells for desmin, myogenin, and CD56. Ki67 score was 10-12%, and WT-1 was negative in the epithelial component suggestive of RMS. The integrity of Gerota’s fascia was maintained. Renal vascular and ureteric margins were free of tumor. There was no lymphovascular invasion. Heterogeneous elements such as skeletal muscle, rhabdomyoblastic differentiation, and squamous elements were seen. Chemotherapy-induced changes with stromal maturation and rhabdoid differentiation were seen.

## Discussion

The presentation of renal RMS in pediatric patients is exceedingly rare, with only a limited number of cases documented in the literature. While RMS predominantly arises in the head and neck region, particularly the orbit, and less commonly in the genitourinary tract, renal involvement is a rare occurrence [6]. The rarity of renal RMS poses diagnostic challenges due to its atypical clinical presentation and radiological features. Our case adds to the sparse literature on this entity and underscores the importance of considering RMS in the differential diagnosis of pediatric renal masses.

The treatment approach for pediatric RMS has been standardized by the Children’s Oncology Group protocol, encompassing a multimodal strategy involving surgery, chemotherapy, and radiotherapy [6]. This approach, tailored for pediatric patients, has also been widely adopted in the management of adult RMS. The implementation of multimodality treatment has significantly improved survival outcomes, with reported cure rates ranging from 70% to 90% in pediatric RMS cases [7-9].

However, despite advancements in treatment modalities, certain prognostic factors continue to influence outcomes. Older age at diagnosis has been consistently associated with worse prognosis in pediatric trials, suggesting that pediatric and adult RMS may have distinct clinical behaviors and responses to therapy [10]. Unfavorable prognostic variables, such as the alveolar subtype and regional or distant spread, are more frequently observed in adults than in children, further emphasizing the importance of tailoring treatment strategies to the age and clinical characteristics of the patient [10].

Histopathologically, the majority of reported cases of renal RMS exhibit the embryonal subtype, with a smaller proportion characterized as a pleomorphic subtype. Alveolar RMS, although rare in renal involvement, has been documented in a limited number of cases [9]. These tumors typically arise from the renal cortex and predominantly affect pediatric patients, with the peak incidence occurring between two and six years of age, and approximately 75% of cases diagnosed before the age of five [11].

While the presence of rhabdomyoblasts is a hallmark histological feature of RMS, their absence, particularly in poorly differentiated tumors, can complicate diagnosis. In such cases, histochemical markers specific to muscle cells (e.g. desmin, myogenin, MyoD1, and muscle actin) play a crucial role in confirming the diagnosis [12]. The clinical presentation of renal RMS varies widely, ranging from asymptomatic cases incidentally discovered on imaging studies to those causing pressure symptoms or manifesting classic tumor-related symptoms such as abdominal pain, hematuria, and palpable mass [13].

Our case underscores the need for continued research into the molecular mechanisms underlying renal RMS pathogenesis and progression. It elucidates the genetic alterations driving tumor development and may identify novel therapeutic targets and inform the development of more effective treatment strategies. Collaborative efforts to establish standardized diagnostic criteria and treatment guidelines for renal RMS are imperative to optimize patient outcomes. Multicenter studies involving larger patient cohorts are warranted to delineate the clinical characteristics, prognostic factors, and treatment outcomes of renal RMS comprehensively. Moreover, long-term follow-up studies are essential to assess disease recurrence rates and long-term survival outcomes in pediatric patients with renal RMS.

The management of renal RMS remains challenging due to its rarity and diverse clinical presentations. Further research is warranted to elucidate the molecular mechanisms underlying tumor development and progression, with the ultimate goal of refining diagnostic and therapeutic strategies to optimize patient outcomes.

## Conclusions

This case report highlights the considerable difficulties involved in detecting and treating RMS that originates from the upper pole of the kidney in a pediatric patient. Given the uncommon occurrence of this presentation, it is crucial to maintain a heightened level of suspicion for RMS while considering potential causes of kidney masses in children, even in unusual sites. The significance of our experience underscores the importance of employing a comprehensive approach that incorporates various methods such as imaging examinations, histological evaluation, and intensive treatment tactics such as surgery, chemotherapy, and maybe radiotherapy, based on individual risk factors. Although the prognosis for renal RMS is uncertain, developments in multimodal treatment provide optimism for better patient outcomes. Additional investigation is essential to clarify the precise molecular pathways that are responsible for this uncommon condition and to discover potential new targets for therapy. Furthermore, it is necessary to engage in collaborative endeavors to develop uniform diagnostic criteria and treatment guidelines for renal RMS to enhance the quality of patient care.
